# Learning Games: A New Tool for Orthodontic Education

**DOI:** 10.3390/ijerph20032039

**Published:** 2023-01-22

**Authors:** Edmund Khoo, Austin Le, Mitchell J. Lipp

**Affiliations:** 1 Indiana University School of Dentistry, Indianapolis, IN 46202, USA; 2 Eastman Institute of Oral Health, University of Rochester, Rochester, NY 14602, USA; 3 Department of Population Health, New York University Grossman School of Medicine, New York, NY 10016, USA; 4 Department of Orthodontics, New York University College of Dentistry, New York, NY 10010, USA

**Keywords:** orthodontics, dental education, learning games, teaching

## Abstract

Learning games that are based on current scientific concepts are underutilized in dental education. This paper explores the relevant science of learning and discusses several principles that are conducive to learning and teaching in an educational setting, namely retrieval practice, feedback, motivation, and engagement. A discussion of learning games in health professional education ensues, followed by a description of relevant best practices in game design for learning. This paper concludes by presenting Dealodontics©, a card game developed at New York University College of Dentistry with the goal of helping second-year dental students review, practice, and apply basic skills relevant to their orthodontics competency requirements.

## 1. Introduction

Higher education—including dental education—has largely overlooked over a century’s worth of scientific study and knowledge on the principles of teaching and learning [1,2,3,4,5,6,7]. Learning games that are based upon current scientific concepts have been developed and tested [8,9,10], representing a potentially underutilized means for advancing education in dentistry. This paper will explore the relevant science of learning, provide examples of learning games in health professional education, establish best practices in game design, and present Dealodontics©—a card game developed at New York University (NYU) College of Dentistry with the goal of helping second-year dental (D2) students review, practice, and apply basic skills relevant to orthodontic competency.

## 2. The Science of Learning

Several detailed overviews of the principles underpinning the science of learning can be found in the literature [5,10,11]. Key principles relevant to teaching and learning include retrieval practice, feedback, motivation, and engagement.

### 2.1. Retrieval Practice

Decades of research reveal that retrieving knowledge strengthens and improves memory [11,12]. Some researchers have coined the term “retrieval-based learning” to label the phenomenon in which practicing active retrieval enhances long-term meaningful learning [13]. Retrieval practice can be thought of as the act of bringing learned information from long-term memory to mind, which is a process that requires effort. While tests and exams are traditionally used for the purposes of assessing knowledge, tests actually reinforce knowledge and improve memory. This is sometimes referred to as the “testing effect” [10]. Student learning can be guided by low- or no-stakes tests (i.e., formative assessments), which can ultimately affect performance outcomes on final tests (i.e., summative assessments). For example, in an experiment that applied retrieval practice to a classroom setting, researchers demonstrated that students performed better (based on a final exam) on topics that had been quizzed beforehand than those that were not [14]. Moreover, the benefits of quizzing extended to items that required transfer of knowledge, which is to say, items that were not exactly identical to the ones quizzed in class [15]. How we test and how we subsequently assess performance are inextricably linked since the way we test shapes the learning environment [4].

Cognitive science research suggests that the act of remembering in order to generate a response will strengthen the memory trace such that the just-retrieved information is more likely to be retrieved (or less likely to be forgotten) in the future [10]. Furthermore, retrieval-based learning has been shown to lead to better final test scores than other approaches such as concept mapping [16,17]. However, an important consideration that instructors and educators should keep in mind is that the benefits of retrieval practice seem to depend, at least to some extent, on successful retrieval outcomes [11]. In other words, if students are experiencing very low success during their retrieval practice exercises, they are unlikely to see improvements in memory [12]. This has important implications for the interplay between difficulty and success rate during the design of retrieval practice exercises. Appreciating the importance of retrieval practice as a powerful and effective method to advance learning should be a priority for educators who may otherwise stagnate within the traditional approaches of passive learning, such as unidirectional presentations, conventional lectures, or generic videos.

### 2.2. Feedback

Feedback can be an important component of effective learning, but in and of itself does not universally guarantee improved learning outcomes. While systematic reviews and meta-analyses of the literature on feedback conclude that, on the whole, feedback enhances student learning [18,19], the findings are notably heterogeneous, with numerous studies demonstrating that not all types of feedback are beneficial and that some may even damage self-efficacy [20,21,22]. In a study in which dental students evaluated different types of feedback, researchers found that corrective feedback given with descriptive written feedback was more beneficial to students’ attitudes towards instruction and self-confidence in ability than corrective feedback with pass/fail grades or emoticons [23]. The authors conclude that in learning, feedback should be based upon conditions that foster safety, trust, engagement, motivation, and commitment toward achieving the goal. In general, feedback is important insofar as it provides information to the learner about his or her own performance or how to improve, without jeopardizing his or her self-efficacy.

Feedback can also be utilized to overcome certain problems directly associated with retrieval-based learning. For example, it was mentioned earlier that the positive memory-related benefits of retrieval practice may not be observed if the success of retrieval is low during practice. One demonstrable means of addressing said situation is to provide feedback, including the correct response after an unsuccessful attempt [12,24].

Research further suggests that more frequent feedback is important early on during the learning processes [25]. The length between feedback intervals can then be increased as learning progresses, so that learners can better self-evaluate their performance and not become overly reliant on external sources of feedback [10].

### 2.3. Motivation

Motivation can be broadly categorized into extrinsic motivation (“carrot and stick”) and intrinsic motivation (self-directed) [26,27]. Intrinsic motivation, particularly in learning, is believed to be better insofar as educators strive to develop life-long learners [28,29]. While an exhaustive discussion of motivation is beyond the scope of the present paper, understanding the key aspects of the self-determination theory can be useful for developing strategies in learning and education to facilitate intrinsic motivation among students. Self-determination, as theorized by Ryan and Deci [30], posits that three human psychological needs must be fulfilled in order for individuals to be intrinsically motivated for optimal functioning: competence, autonomy, and relatedness.

Competence refers to the ability for an individual to feel some level of effectiveness and confidence in what they do [31]. This is related not only to what is innate to a given individual, but also to the impact of the environment on said individual. Individuals who are on a positive track to achieving competence and are in positive environments that encourage the achievement of competence tend to have higher levels of intrinsic motivation.

Autonomy describes the ability of an individual to function by self-regulation. In its purest form, autonomous function is self-organized and done of one’s own volition. Like competence, the social environment also has a significant impact on developing autonomy. When individuals are in micro-managed environments, for example, they risk losing some degree of autonomy. Conversely, if an individual is in an environment in which proactiveness is appreciated and freedom of decision-making is encouraged, autonomy can flourish [31].

Relatedness refers to a sense of belonging within a group and an attachment to others in said group and is typically fostered when an individual feels included as being “a key part of the group”. This, in turn, promotes the internalization of learnt values and skills. An environment that is warm and inviting is conducive to relatedness, whereas one that is cold and dissociative is antithetical to relatedness.

### 2.4. Engagement

Student engagement, as described by the Glossary of Education Reform, refers to the degree of attention, curiosity, interest, optimism, and passion that students show when they are learning or being taught [32]. To some extent, student engagement is inextricably related to the level of motivation they have to learn and progress with their education. However, it is certainly important for educators to distinguish between engagement and compliance. Measures of attendance, requirements for completing work, test scores, and grades, for example, are veritably about incentivizing compliance at their core. Genuine engagement, in contrast, occurs when students are operating on a deeper level, such that engaged students tend to be self-motivated, self-directed, inquisitive, and inclined to make connections to prior learning [33].

Engagement is a complex topic that may be affected by an array of factors, such as gender, age, background, socioeconomic status, generational factors, the learning environment, and group size [34,35,36,37]. Furthermore, a major challenge in fostering student engagement is identifying the appropriate level of difficulty for individual students. These challenges may be exacerbated in large academic institutions in which it can prove difficult to individualize teaching. Educators across all levels have grappled with methods to increase student engagement. One example comes from the Universal Design for Learning (UDL) group, which put forth a set of guidelines that were intended to serve as concrete suggestions that could be applied to learning in any discipline [33]. Although originally developed for K-12, these guidelines are consistent with the needs of the adult learner as they encompass key aspects of internal motivation, task relevance, active experiences, and self-direction [33,38]. Seven suggestions for improving student engagement are outlined below.

- Make Learning Relevant. Students need to be reminded of the clinical relevance for what is being taught and how it relates to the job.
- Remove Barriers to Learning. For example, consider students with language barriers. Perhaps content can be presented in alternative formats, utilizing image-rich modalities.
- Include Choice and Voice. Students value control of their learning. Provide options and opportunities for students to make decisions. This may also be an opportunity for students to identify appropriate challenges. Choice also ensures that students are not passive learners.
- Create a Safe Space for Learning. Provide clear expectations without threats or distractions. Encourage students to take risks and even fail. Failure is an opportunity to reflect on what went wrong and what could be done differently next time.
- Encourage Social Learning. Encourage peer collaborations and teamwork. Creating a community of learners in the classroom prepares students to work in teams.
- Make Feedback Matter. Feedback should be meaningful, frequent, timely, specific, and sensitive to the individual.
- Set Learning Goals. Let students know the goal they are working toward. If the goal is a long-term objective, break it into smaller achievable steps so that students are not overwhelmed.

## 3. Serious (Learning) Games

### 3.1. Serious Games

Many of the cognitive principles underpinning the science of learning are well-suited to games. “Serious games”, a term first coined several decades ago [39], refers to games designed specifically for non-entertainment purposes, with applications in fields such as the military, education, and business [40,41]. Interest in learning games in education has burgeoned over the past decade [10,42,43,44,45].

Serious games can be designed to foster an immersive environment for students, allowing learners to interact and engage with the material in a manner distinct from traditional study methods. Researchers have demonstrated that serious games in educational settings may increase the likelihood of providing students with authentic learning experiences [46], increase their motivation for learning [47], improve task engagement [41], and facilitate knowledge acquisition [48].

Several recent studies have investigated the effectiveness of serious games in education [45]. For example, a systematic review of serious games applied in higher education found that games and simulations had a positive effect on learning goals [49]. Similarly, a large meta-analysis of studies investigating the impact of serious games on science learning concluded that serious games were found to be more beneficial than conventional teaching methods on three learning outcomes: declarative knowledge, knowledge retention, and procedural knowledge [50].

### 3.2. Learning (Serious) Games in Professional Health Education

The use of gaming in health education is not new, and research has shown that the application of serious games in health education, including dental education, likewise has the potential to have a positive impact on learning and motivation [51,52]. While learning games on their own can have a similar impact on learning outcomes as other teaching modalities, they can also be employed as a helpful supplement to traditional modalities of teaching in order to enhance student engagement and learning [53].

Some benefits of implementing learning games in healthcare and dental education include but are not limited to: (1) increasing engagement and motivation; (2) providing feedback, peer assessment, and interaction; (3) creating an environment for experiential self-paced learning; (4) providing a safe virtual environment with no harm to actual live patients; and (5) improving inter-professional relationships between the student and their teachers and patients. However, it is worth noting that some research suggests that the use of learning games education may promote a competitive environment, causing some learners to feel threatened or intimidated [54]. In addition, poorly designed or dated gaming technology may also generate a sense of boredom, which is counterproductive to engagement and motivation [55]. Therefore, educators must be aware that incorporating learning games into educational curricula is not a panacea for all learning woes. For a serious game to succeed in promoting engagement, motivation, and learning outcomes, it must succeed both as a game and as an instructional tool. A serious game for learning must be consistent with the science of learning and best practices for game design.

### 3.3. Best Practices for Learning Games

In general, the primary purpose of playing games is to have fun. Learning, however, may be effortful and demanding. The key to both effective instruction and designing a learning game is to find an appropriate level of challenge for the student. If the challenge is exceedingly difficult, the student may get frustrated, lose interest, or quit. If the challenge is too easy, the game may not be fun and learning may not occur. An important consideration is that the learning game should support—not distract from—learning. Eleven of the best practices for designing learning games, adapted from a field book on the gamification of learning [56], are described below and in Table 1. These practices support the four important aforementioned principles underlying the science of learning.

- **Practice 1**—Design the learning game to meet specific instructional objectives.
- **Practice 2**—Embed the learning game into a curriculum.
- **Practice 3**—Keep rules, scoring, and leveling simple.
- **Practice 4**—Get learners comfortable with the rules and gameplay before they start.
- **Practice 5**—Do not focus the game on “winning” only. Focus on learning outcomes.
- **Practice 6**—If possible, create the game so learners must work in groups.
- **Practice 7**—The cognitive activities in the game should match the cognitive activities on the job. The closer the two match, the better the learning transfer.
- **Practice 8**—Plan for re-playability.
- **Practice 9**—Make the game interactive and focus on player activities.
- **Practice 10**—Decide how you are going to measure the effectiveness of the game before you design it.
- **Practice 11**—Winning should primarily be a result of knowledge acquisition or creation.

**Table 1:** Self-Evaluation of Dealodontics relative to Best Practices for Learning Game (Adapted from “The Gamification of Learning and Instruction Fieldbook”) [56].

Best Practices for Learning Games	Dealodontics	Description
1—Design the game to meet specific instructional objectives.	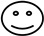	Objectives are explicitly stated in each session of the course
2—Embed the game into a curriculum.	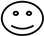	Embedded in a three-year, competency-based curricular track
3—Keep rules, scoring, and leveling simple.	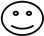	Rules, scoring, look, and feel of the game are familiar and simple
4—Get learners comfortable with the rules/gameplay before playing	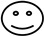	Pre-Game: 15 min PowerPoint overview of rules/gameplay
5—Do not focus on “winning” only. Focus on learning outcomes.	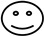	Game reflects learning objectives. No stakes beyond winning
6—Create the game so learners must work in groups.	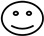	Teams of two players
7—The cognitive activities in the game should match the job.	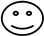	Game is clinically relevant to general dental practice
8—Plan for re-playability.	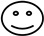	Students ask to play again
9—Make the game interactive and focus on player activities.	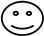	Gameplay is active and interactive
10—Decide how to measure effectiveness before you design it.	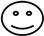	Students are surveyed after playing the game
11—Winning should primarily be a result of learning outcomes.	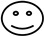	Points are accrued by successfully answering questions. Students have influence over the difficulty of questions they receive

## 4. Dealodontics©: A Learning Card Game for Orthodontics

### 4.1. Description of Dealodontics©

In 2016, at NYU College of Dentistry, a card game was created for dental students to reinforce and apply basic orthodontic concepts. This game, Dealodontics©, is built upon the learning objectives for the second-year (D2) orthodontics course and its required competencies for the diagnosis and management of malocclusion/skeletal problems and space management. Dealodontics© incorporates clinical photographs and questions to assess students’ skills in diagnosis and patient management. The game was designed to be an enjoyable, interactive, and collaborative learning experience, supplementing competency-based instruction in the curriculum.

Dental students played Dealodontics© as part of the laboratory portion of their D2 course in Orthodontics. The game follows a series of didactic presentations and lab experiences, in which students are introduced to the terms and concepts that appear in the game. Before the game, there is a 15 min overview of the rules and gameplay, followed by a pre-game warm-up round using flashcards. By reviewing foundational skills before playing the game, students are better prepared, more comfortable, and more confident in managing challenges during gameplay.

Gameplay is moderated by a “dealer”—typically an orthodontic instructor or orthodontic post-graduate (PG) student. The dealer is responsible for moderating the pre-game warm-up and monitors flow during the game by determining the order of turns; moderating questions, hints, and responses; timing; scoring; regulating inter-team exchanges; and collecting team cards.

Students play in teams comprising two or more players. This reduces frustration and allows for communication between students to address problems, discuss varying perspectives, and work collaboratively as a team toward a common goal (to accumulate enough points to win the game). Game group sizes are limited to three teams (two students per team) and one dealer. In a typical D2 lab session, 10–15 games are played simultaneously, suggesting the scalable nature of Dealodontics© for larger institutions.

The game itself consists of three decks of cards (flashcards for the pre-game warm-up, game cards, and team cards), a one-minute timer, and score sheets. The goal of the game is to be the first team to accrue 32 points. Teams score points by correctly answering questions based on images, chiefly intraoral photos or radiographs. Looking at the images, the teams choose one question based on categories (Beginner, Advanced, Expert) of varying difficulty corresponding to points (2, 3, and 4 points, respectively). During a turn, teams may ask for a hint if they consequently accept a one-point deduction.

**Figure 1:**
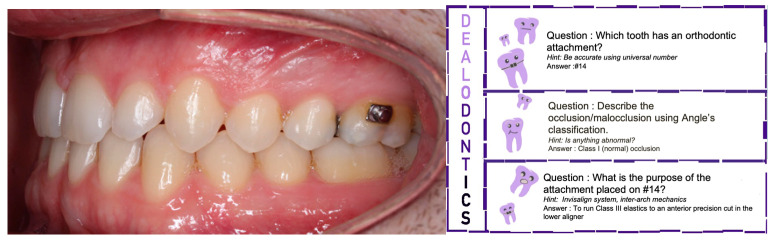
Sample game card used in playing Dealodontics© Teams only see the image. The reverse side with questions of increasing difficulty is seen by the dealer.

Figure 1 presents an example of a typical game card. Only the dealers possess the game cards and can read the side of the card with the text (questions, hints, answers). Teams have one minute to answer their question.

**Figure 2:**
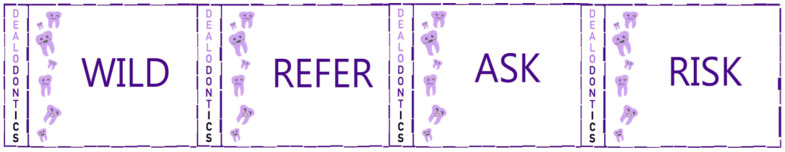
There are four types of team cards.

Teams are encouraged to use their team cards (Figure 2) during the game in order to maximize the potential accrual of points. At the beginning of the game, each team receives seven team cards. These cards can be played by the team during their turn at their discretion and can only be used once during the game. This introduces a strategical element as well as a factor of randomness to the gameplay. There are four types of team cards:

- RISK: Challenge the previous team’s answer. (If correct, the team get points, but if not, the team loses a turn).
- REFER: Pass and get a new game card without penalty.
- ASK: Receive a “free” hint (no point deduction).
- WILD: Get an extra turn.

### 4.2. Evaluation of Dealodontics©

Dealodontics© was designed to be consistent with the current cognitive science research and aforementioned principles of learning as well as best practices in learning game design, as illustrated in Table 1. Students retrieve, apply, and practice content and skills previously introduced in the D2 course. Immediate verbal feedback occurs within and between teams, as well as between teams and dealers. Through active gameplay, students are engaged by extrinsic motivators (e.g., wanting to win) and by intrinsic motivators (e.g., knowing that the acquired knowledge is clinically relevant to dentists). Motivation is further enhanced by contextualizing gameplay in a safe “no stakes” environment in which the students can feel free to take risks. The game actually encourages students to take risks as part of a strategy to win by accruing more points, while playing team cards gives students a greater sense of autonomy during gameplay. Working collaboratively reduces student anxiety about being individually evaluated and encourages an open dialogue and problem-solving among team players.

### 4.3. Reception of Dealodontics© among Students

**Figure 3:**
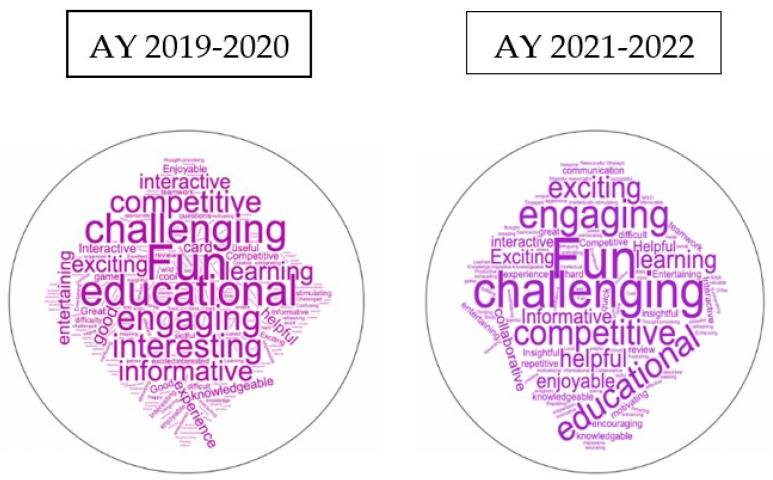
Sample word clouds. Word clouds demonstrate words used by students based on frequency describing the gameplaying experience (without prompts). AY 2019–2020 Word Cloud: 239 D2 students responded (RR = 66%). Most reported words were: 64% “fun”, 17% “educational”, 15% “engaging”, and 13% “challenging”. AY 2021–2022 Word Cloud: 115 D2 students responded (RR = 47%). Most reported words were: 54% “fun”, 25% “challenging”, 13% “engaging”, and 10% “educational”. “Fun” was the most commonly occurring word in all years surveyed.

After playing the game, the students were asked to complete a voluntary anonymous online survey. The intent of the survey was to identify areas to improve the game. The findings of this survey have been presented elsewhere [57]. Briefly, the one area that appeared to be problematic was the flow of the game. In response to this feedback, modifications were made to improve the flow. For example, fewer teams per game group were adopted, and the time limit for responses was decreased to one minute in order to speed up the game pace. Generally, the gameplay experience was positive in all other areas (competence, positive effects, immersion, challenge, and learning) and not negative (tension/annoyance, negative effects). At the end of the survey, there was an open-ended question permitting students to list three words to describe their game experience (Figure 3). In all the years the survey was given, the most commonly reported word was “fun” [57], which hopefully speaks to the level of engagement that the students felt while playing. As a result of this consistent positive response, Dealodontics© is used routinely in the predoctoral orthodontic education at NYU College of Dentistry, the largest dental school in the US. Although there are no current plans to commercialize Dealodontics©, we believe that such education and learning games have promising potential and may even be utilized among digital platforms for ease of dissemination and use.

## 5. Conclusions

The present paper explored recent information from areas well beyond dentistry and orthodontics, including cognitive psychology and education. Despite advancements in the science of teaching and learning, it is surprising that many educational programs have not adapted their pedagogical methods. Effective instruction for the adult learner, such as those in dental education, should be meaningful, relevant, active, and interactive. Methods of instruction and assessment should be varied, yet with consistent explicit objectives and criteria. Learning can be difficult and effortful, but it need not be dreadful. Learning games provide an alternative method in which students can practice and learn while potentially having fun as well. Dealodontics© was specifically designed for Orthodontics and emphasizes clinical cognitive skills, including retrieval practice, engagement, analysis, and problem-solving, that are relative to competence at the predoctoral level. Professional observers, videos, and surveys provide evidence to support the notion that students are engaged and enjoying the gameplay. The communication and interplay that occur during gameplay are notable. Educators must move beyond the traditional approach involving passive presentations which are suboptimal for teaching and learning. New instructional methods should regularly consider retrieval practice, feedback, motivation, and engagement. Perhaps most importantly, learning outcomes and student perceptions must be continually monitored to optimize the learning experience.

## Data Availability

The data that support the findings of this study are available from the corresponding author upon reasonable request.
